# Testing possible causes of gametocyte reduction in temporally out-of-synch malaria infections

**DOI:** 10.1186/s12936-020-3107-1

**Published:** 2020-01-14

**Authors:** Mary L. Westwood, Aidan J. O’Donnell, Petra Schneider, Gregory F. Albery, Kimberley F. Prior, Sarah E. Reece

**Affiliations:** 1 Institute of Evolutionary Biology and Institute of Immunology and Infection Research, School of Biological Sciences, University of Edinburgh, Charlotte Auerbach Road, Edinburgh, EH9 3FL UK; 2 Department of Biology, Georgetown University, 37th and O Streets NW, Washington, DC 20057 USA

**Keywords:** Malaria, *Plasmodium*, Innate immunity, TNF-α, Reproductive effort, Phenotypic plasticity, Chronoimmunology, Inflammatory cytokine, Intraerythrocytic development cycle, Conversion rate

## Abstract

**Background:**

The intraerythrocytic development cycle (IDC) of the rodent malaria *Plasmodium chabaudi* is coordinated with host circadian rhythms. When this coordination is disrupted, parasites suffer a 50% reduction in both asexual stages and sexual stage gametocytes over the acute phase of infection. Reduced gametocyte density may not simply follow from a loss of asexuals because investment into gametocytes (“conversion rate”) is a plastic trait; furthermore, the densities of both asexuals and gametocytes are highly dynamic during infection. Hence, the reasons for the reduction of gametocytes in infections that are out-of-synch with host circadian rhythms remain unclear. Here, two explanations are tested: first, whether out-of-synch parasites reduce their conversion rate to prioritize asexual replication via reproductive restraint; second, whether out-of-synch gametocytes experience elevated clearance by the host’s circadian immune responses.

**Methods:**

First, conversion rate data were analysed from a previous experiment comparing infections of *P. chabaudi* that were in-synch or 12 h out-of-synch with host circadian rhythms. Second, three new experiments examined whether the inflammatory cytokine TNF varies in its gametocytocidal efficacy according to host time-of-day and gametocyte age.

**Results:**

There was no evidence that parasites reduce conversion or that their gametocytes become more vulnerable to TNF when out-of-synch with host circadian rhythms.

**Conclusions:**

The factors causing the reduction of gametocytes in out-of-synch infections remain mysterious. Candidates for future investigation include alternative rhythmic factors involved in innate immune responses and the rhythmicity in essential resources required for gametocyte development. Explaining why it matters for gametocytes to be synchronized to host circadian rhythms might suggest novel approaches to blocking transmission.

## Background

A hallmark of many species of malaria (*Plasmodium*) parasite is synchronous, rhythmic development during asexual replication cycles within host red blood cells. For *Plasmodium chabaudi*, each intraerythrocytic development cycle (IDC) spans 24 h, at the end of which mature parasites burst to release their merozoite progeny. Each merozoite is committed to either asexual replication or to differentiating into a sexual stage (gametocyte) for transmission to mosquitoes. Like the human malaria *Plasmodium falciparum,* the asexual development of the rodent malaria *P. chabaudi* progresses through sequential stages within the IDC in synchrony with each other, transitioning between IDC stages at particular times-of-day [1, 2].

Though melatonin was previously suggested to be a cue for the timing of the IDC [3], new experiments reveal the IDC schedule is determined by the timing of the host’s feeding rhythm. Specifically, it was found that IDC completion (schizogony) switches from the night (dark phase) to the daytime when hosts only have access to food in the daytime (light phase) [4, 5].

Maintaining coordination with host circadian rhythms appears important to parasites. If infections are initiated such that early IDC stages are inoculated into the host in the evening (12 h out-of-synch with the host rhythm) rather than in the early morning (when they usually occur; in-synch), parasites suffer a 50% reduction of both asexually replicating stages and gametocytes across the acute phase [1, 6]. Asexual stages become increasingly demanding of host resources as they progress through the IDC. If these resources appear in the blood in a circadian manner, asexual parasites that are out-of-synch with host rhythms may be unable to fulfil their needs and die, or have to pause development until resources are available [7]. Either death or a delay to replication could explain why asexual density is lower in out-of-synch infections [8]. However, accounting for the reduction of gametocytes in out-of-synch infections is more complex. Intuitively, the reduction in the density of asexual stages might be expected to translate directly into an equal reduction in gametocyte density. However, investment in gametocytes (the proportion of asexuals in a given IDC cohort that produce gametocyte-committed progeny; “conversion rate”) is a plastic trait that varies considerably during infections [9, 10]. Furthermore, given the different developmental durations and lifespans of asexuals and gametocytes, and their rapidly changing densities during infections, close correlation between asexual and gametocyte densities is unusual. Instead, the reduction in gametocyte density in infections that are out-of-synch with the host’s circadian rhythms could be explained by either (or both) a “parasite strategy” to promote within-host survival, or increased host-mediated removal of out-of-synch gametocytes from circulation.

The “parasite strategy” scenario stems from a body of work revealing that malaria parasites adjust their conversion rate in response to changes in the within-host environment in ways that maximise their fitness [9, 11–14]. Specifically, under stressful conditions, parasites reduce conversion by adopting reproductive restraint and investing more in survival [10]. However, under extremely stressful conditions (when the infection is at risk of being cleared by the host immune system or drugs), parasites increase conversion, producing mostly transmission stages (gametocytes) and thus making a terminal investment [10]. Reproductive restraint enables more parasites to be allocated to asexual replication, which equips the parasite with “safety in numbers” to withstand within-host stressors. The loss of short-term transmission potential that results from reproductive restraint is compensated for by the improved prospects for within-host survival and future transmission opportunities [10]. Such reproductive restraint has been observed in both *P. chabaudi* and *P. falciparum* in response to treatment with low doses of anti-malarial drugs and within-host competition [10, 12, 15–18]. Thus, parasites may interpret the reduction in asexual density caused by being out-of-synch to the host’s rhythm as a situation in which reproductive restraint is beneficial to them. Therefore, parasites in out-of-synch infections are expected to reduce conversion, at least during the first few IDCs when asexual densities are most affected by being out-of-synch.

Alternatively (or additionally), the host’s circadian immune responses could be more effective at removing out-of-synch gametocytes from circulation. This “immune killing” hypothesis requires that: (i) the appearance of a gametocytocidal immune factor in the blood follows a circadian rhythm set by the host’s circadian clock; (ii) the vulnerability of gametocytes varies throughout their development, such that during in-synch infections, gametocytes are at a less vulnerable age when the immune factor appears or peaks, and so, a more vulnerable age coincides with the immune factor in out-of-synch infections; and (iii) the gametocytocidal factor is part of the innate immune response because the costs of being out-of-synch occur in the first few days of infection when primarily innate responses are active. The only gametocytocidal factor reported to rapidly clear *P. chabaudi* gametocytes from the blood is the pro-inflammatory cytokine tumour necrosis factor (TNF) [19]. When the host’s TNF receptor is blocked, the gametocyte density of *P. chabaudi*-infected mice increases (on average by 44%), regardless of parasite clone and asexual parasite density [19]. This increase occurs within 24 h which is too soon for mature gametocytes produced via an increase in conversion rate to be detected, and the rate of gametocytogenesis was not affected by the TNF receptor blockade, implying that gametocyte survival was improved in the absence of TNF [19]. Asexual stages are also vulnerable to TNF which acts on, for example, *P. falciparum* via a calcium-cAMP downstream signalling, with PCNA1 (proliferating‐cell nuclear antigen-1) as a possible target [20]. Whether a similar mechanism also operates in gametocytes and could mediate age-specific vulnerability to TNF is unknown. TNF expression is rhythmic in mice and generally peaks during the resting phase—i.e. during the day [21]. However, standing rhythms in inflammation may be altered by infection: in *P. chabaudi* infected mice, rhythmicity in TNF is also linked to the time-of-day that schizogony occurs [4]. Further complexity in TNF-α rhythms may arise from host rhythms of TNF-α production and decay, induction of TNF expression in response to schizogony, and possibly from time-of-day-dependent activities of the innate immune cells that TNF stimulates. Therefore, it is hard to predict the time-of-day (i.e. age) at which gametocytes are most vulnerable, or exposed, to TNF.

Here, both the “parasite strategy” (conversion rate modulation) and “immune killing” hypotheses were investigated using *P. chabaudi*. First, conversion was estimated for a previously collected dataset in which parasites were either in-synch or out-of-synch with host rhythms. Conversion rates were estimated using a method for statistical inference which follows each time-series of within-host infection dynamics, including the densities of asexual parasites, RBCs, and the starting gametocyte density for each infection [10, 22]. Next, the immune killing hypothesis was examined using a series of three experiments. The first tested whether host circadian rhythms affect the clearance of TNF from the blood. The second and third experiments examined whether TNF differentially affects gametocyte survival at different times of day, using wild type (WT) and clock mutant mice, respectively. Unraveling the cause of gametocyte reduction in temporally desynchronised infections will further the understanding of the causes and consequences of rhythmicity in the IDC. This knowledge could guide the development of novel anti-malarial treatments, and may inform predictions for the proximate and ultimate responses of parasites to temporal shifts in vector behaviour caused by the widespread usage of insecticide-treated bed nets.

## Methods

### Testing the parasite strategy hypothesis

#### Do parasites in out-of-synch infections reduce investment into conversion?

A previously published data set [6] was used to compare conversion rates at the start of infections for parasites that were in- and out-of-synch with host rhythms. Briefly, *P. chabaudi* strain AJ ring stage parasites (1 × 10^6^ parasitized RBCs) were harvested from donor mice kept in standard (12-h light: 12-h dark) or reversed (12-h dark: 12-h light) lighting schedules and used to infect recipient mice (10- to 12-week-old male MF1) in the same lighting schedule as their donor mice, or into mice kept under the opposite (reversed) lighting schedule. Thus, parasites in recipient hosts kept in the same lighting schedule as the donor host remained in synchrony or “in-synch” with the host circadian rhythm and parasites moved between lighting schedules became 12-h “out-of-synch”. This produced four groups of infections: two in-synch and two out-of-synch. Data were collected for days 0–7 post-treatment; blood samples to quantify gametocyte densities (10 μL) were taken every day and total parasite densities (5 μL) were taken on days 1, 3, 5, and 7. DNA and RNA were extracted as described in Schneider et al. [10]. Total parasite densities were quantified by qPCR (quantitative polymerase chain reaction) and gametocytes by RT-qPCR (reverse-transcriptase qPCR), both targeting the CG2 gene (PCHAS_0620900, previously named PC302249.00.0) [23]. Asexual parasite density was calculated by subtracting gametocyte numbers from total parasite density. Red blood cell (RBC) densities were measured using flow cytometry (Beckman Coulter) every day.

Unlike previous methods which made unrealistic assumptions about infections (such as fixed conversion during maturation of sexual stages; equal death rates for asexuals and gametocytes; short survival of gametocytes and thus, non-overlapping cohorts of gametocytes), the method used here more realistically infers conversion in dynamic infections [22]. The method requires at least 7 days of continuous data, including daily RBC, asexual, and gametocyte densities for each infection. Therefore, to provide daily estimates of asexual density, missing values were interpolated between sequential data points by taking the mean of the preceding and subsequent day. These autocorrelated data resulted in an average estimate of constant conversion throughout each infection. These average conversion rates were compared between in-synch and out-of-synch parasites, for a data set including all infections, and a dataset comprising the subset of infections which met strict criteria for model fitting. Infections are excluded if the residuals showed a significant relationship to natural logged densities of gametocytes, and/or if less than four of the five candidate splines could be fitted [10, 22]. This resulted in the exclusion of 1 in-synch infection and 7 out-of-synch infections (out of 12 infections per each treatment; random subsets of 5 in-synch infections resulted in qualitatively similar analysis outcomes).

### Testing the immune killing hypothesis

Three experiments were conducted to test: (i) whether changes in the concentration of TNF vary by time-of-day of injection and dose (which also informed the dose of TNF used in following experiments); (ii) whether gametocyte vulnerability to injected TNF varies according to host time-of-day; (iii) whether gametocyte age mediates vulnerability to injected TNF.

All mice were 6- to 8-week-old male and female WT C57/BL6 mice (in-house supplier, The University of Edinburgh). Mice were provided water containing 0.05% para-aminobenzoic acid (PABA; to enhance parasite growth) and food ad libitum and kept under a standard 12-h light: 12-h dark schedule (except in experiment iii). All experimental mice that were infected received ring stage *P. chabaudi* clone ER parasitized red blood cells (1 × 10^6^) via intraperitoneal (IP) injection.

#### Do changes in concentration of TNF vary by host time-of-day and dose?

Uninfected mice received either 30 μg/kg or 60 μg/kg TNF in 100 μL PBS carrier at either lights on (ZT0, n = 5 for each group) or lights off (ZT12, n = 4 for each group) via IP injection. ZT refers to “Zeitgeber Time” which is defined as the number of hours elapsed since lights on. Two hours post-treatment with TNF, blood was obtained from all mice individually by cardiac puncture and centrifuged to collect plasma for a murine TNF-specific quantitative enzyme-linked immunosorbent assay (ELISA; eBioscience catalog number 88-7324 Mouse TNF alpha ELISA Ready-SET-Go!®).

#### Is gametocyte vulnerability to TNF-dependent on host time-of-day?

To coincide with peak gametocyte density in control infections [10, 24] mice were injected on day 14 PI with either 60 μg/kg TNF dissolved in 100 μL PBS carrier at ZT0 (n = 23) or at ZT12 (n = 17), or with 100 μL PBS carrier at ZT0 (n = 4) or ZT12 (n = 3) via IP injection. RBC densities and thin blood smears were taken from the tail vein of all mice 1-h before TNF-α or control treatment (providing baseline parameter estimates) and also at, 2-, and 12-h post-treatment. The proportion of RBCs containing gametocytes (quantified via microscopy) was multiplied by RBC density to estimate gametocyte density.

#### Is gametocyte vulnerability to TNF dependent on gametocyte age?

**Fig. 1:**
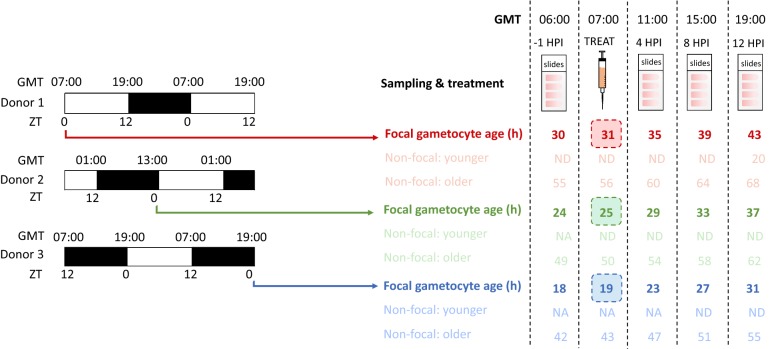
Three photoschedules were used to generate temporally-staggered cohorts of gametocytes simultaneously perturbed at different ages. Parasites were collected from donor mice at their ZT0, allowing infections in experimental mice to be staggered by 6-hours so that at the same times GMT, all infections could be sampled and treated with TNF or PBS yet, different ages of gametocytes (19-, 25-, and 31-hours-old) could be targeted. By using *Per1/2*(−/−) mice housed in constant darkness as the experimental hosts, the relevance of gametocyte age was decoupled from the canonical host circadian-clock-controlled rhythms. The ages of focal gametocyte cohorts (labelled “gametocyte age (h)”, defined as hours post RBC invasion) at each sampling event and treatment (in GMT) are highlighted in bold and the ages of the previous (“younger”) and subsequent (“older”) cohorts are illustrated with faint text. Immature gametocytes not yet detectable via microscopy are denoted by “ND”, and gametocytes not yet produced are denoted by “NA”

Both host-time-of day and gametocyte age co-vary in experiment ii and it is possible these factors oppose the effects of TNF on gametocyte density. Therefore, to focus on gametocyte age without the confounding effect of host rhythms, C57/BL6 *Per1/2* null mice (11- to 22-week-old males and females, mouse line kindly donated by Michael Hastings (MRC Laboratory of Molecular Biology, Cambridge, UK), generated by David Weaver (UMass Medical School, Massachusetts, USA)) were used. These clock mutant have an impaired TTFL clock and exhibit arrhythmic behaviour when placed in constant conditions such as constant darkness [25, 26]. To produce synchronous infections in these arrhythmic mice, wild type C57/BL6 mice were used as parasite donors. Further, to create infections that could be treated with TNF simultaneously (i.e. at the same GMT), yet have focal cohorts of gametocytes at different ages in the experimental mice, staggered infections were set up in the donor mice. This was achieved by offsetting the 12-h light: 12-h dark schedule of each of three donor groups by 6 h (i.e. lights-on, ZT0, at 07.00, 13.00, and 19.00 GMT; Fig. 1).

Under a 12-h light: 12-h dark schedule, a new cohort of gametocytes is produced at each ~ ZT17 [24, 27], translating to 00.00, 06.00, and 12.00 GMT for the three donor groups. Ring stage parasites from these groups were collected at ZT0 to infect experimental mice and produce gametocytes of different ages (31, 25, and 19 h old) at the time (07.00 GMT) of treatment. For example, ring stage parasites collected at ZT0 from donors whose “lights on” is 19:00 GMT produce a cohort of focal gametocytes via schizogony at 12:00 GMT, making these gametocytes 19 h old when treated at 07:00 GMT. Figure 1 illustrates the schedules of the donor mice and their parasites, and the development of the gametocytes produced in the three groups of experimental mice to produce gametocyte cohorts of different ages.

*Plasmodium chabaudi* gametocytes reach maturity between 24 and 36 h after RBC invasion and become identifiable on blood smears between 18 and 24 h old by their morphology and senesce rapidly post-maturation [24]. Although present during treatment and throughout sampling (Fig. 1), younger, non-focal gametocyte cohorts were assumed to make negligible contributions to the observed gametocytes because they are too immature to be detected via microscopy. Assumming a post-maturation half-life of approximately 14 h [24], older, non-focal gametocyte cohorts should be rapidly lost after 38-50 h.

Donor mice were allowed to entrain to their schedules for 1 week prior to infection with *P. chabaudi*, then infections were run until day 7 PI and ring stages were collected to infect the experimental, arrhythmic *Per1/2*(−/−) mice. Parasites begin to lose synchrony in *Per1/2*(−/−) mice after five replication cycles [28], and so, to capture gametocytes while still synchronous yet at quantifiable densities, experimental mice were treated with 125 mg/kg^−1^ phenylhydrazine dissolved in 100 μL PBS (PHZ, promotes gametocytogenesis via anaemia) [11] 4 days prior to infection. Experimental mice were randomly allocated to either the TNF-α or control group with respect to their response to PHZ, measured by RBC density. Then, on day 4 PI, experimental mice were treated with 60 μg/kg TNF-α dissolved in 100 μL PBS carrier (n = 5 for each gametocyte age group, n = 15 total) or 100 μL PBS carrier (n = 4 for each gametocyte age group, n = 12 total). Infections were sampled as per experiment ii to quantify gametocyte densities at − 1 (baseline), 4-, 8-, and 12-h post-injection (HPI) (Fig. 1).

### Data analysis

All analyses were performed using R v. 3.5.1 (R Foundation for Statistical Computing, Vienna, Austria). To meet assumptions of homogeneity and variance, conversion rate estimates were log_10_-transformed. Gametocyte densities for “testing the immune killing hypothesis” parts ii and iii were square root-transformed (in part ii only it was necessary to add half a measurement unit, i.e. 0.5 gametocytes/mL, to all counts to ensure no zero counts). Five outliers were eliminated in “testing the immune killing hypothesis” part ii due to poor fit, and data were scaled in “testing the immune killing hypothesis” parts ii and iii to have a mean of 0 and a standard deviation of 1, post square root-transformation. Linear models were used to analyse conversion rate estimates (testing the conversion hypothesis) and the effects of host time-of-day on TNF concentration (testing the immune killing hypothesis part (i). Linear mixed-effect models were used to analyse gametocyte density (testing the immune killing hypothesis parts (ii) and (iii), using mouse ID as a random effect. To avoid overfitting due to small sample sizes, “Akaike information criterion-corrected” (AICc) values were calculated for each model, and a change in 2 AICc (ΔAICc = 2) was chosen to select the most parsimonious model. Only models directly reflecting the hypotheses under test were fitted.

## Results

### Testing the conversion hypothesis

**Fig. 2:**
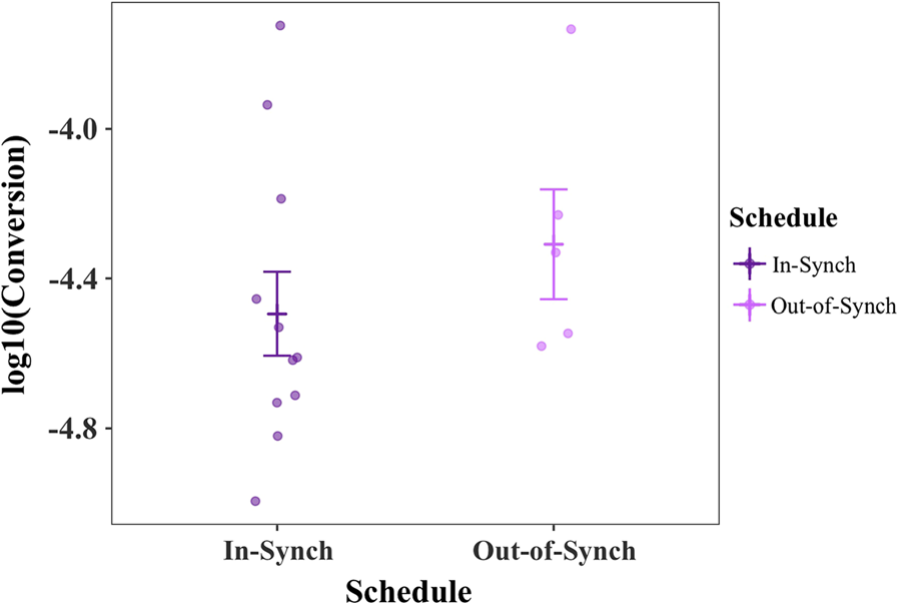
Conversion estimates, alongside mean ± SE (calculated post-transformation), for parasites in- and out-of-synch with host circadian rhythms. Points represent raw data, log_10_-transformed to approximate normality. Data from experiment in O’Donnell et al. [6]

No evidence was found to support the hypothesis that parasites out-of-synch with host rhythms reduce conversion (Table 1; Fig. 2). Conversion rates are best explained by the model containing only “donor photoschedule” (donor mice housed in either the standard, or reversed, light: dark schedule) as a main effect (ΔAICc = 0; Table 1). Specifically, the conversion rate of experimental mice infected with parasites from donors kept in the reversed light: dark schedule was on average 10.4% higher than compared to infections from donors kept in the standard light: dark schedule independent of the light schedule in which the receiving mice were kept. Further, the inclusion of the interaction in the model does not improve model fit (ΔAICc = 6.67, Table 1). Additionally, incorporating parasite schedule (in-synch or out-of-synch with host rhythms) into the most parsimonious model did not improve model fit (ΔAICc = 2.35, Table 1). Further, the model containing only “parasite schedule” returned the least parsimonious fit (ΔAICc = 7.11, Table 1), and has only a 2% chance of being the best approximating model in the given model set (AICc weight = 0.02, Table 1). Supporting a lack of effect of being out-of-synch on conversion rate, the same analysis performed on the full dataset including previously excluded infections also returned no evidence for a difference in conversion rates (Additional file 1).

**Table 1:** Degrees of freedom (df), log-Likelihood (log(*L*)), AICc, ΔAICc (AICc_*model*_ − AICc_*min model*_), and AICc *w* (AICc weight) for each linear model in the conversion analysis ordered in descending fit (best-fitting model at the top)

Model description: Log10 (Conversion) ~	df	log(*L*)	AICc	ΔAICc	AICc *w*
Donor photoschedule	3	− 1.704	11.41	0.000	0.688
Donor photoschedule + parasite schedule	4	− 1.061	13.76	2.351	0.212
Null	2	− 5.764	16.45	5.044	0.055
Donor photoschedule + parasite schedule + donor photoschedule*parasite schedule	5	− 1.037	18.07	6.667	0.025
Schedule	3	− 5.259	18.52	7.110	0.020

The response variable for each model is the log_10_-transformed conversion rate. “Parasite schedule” refers to parasites either in-synch or out-of-synch with the host, and “donor photoschedule” corresponds to parasites taken from donor mice kept in either the standard or reversed light:dark schedule. The null model includes only “mouse” as a random effect

### Testing the immune killing hypothesis

#### Do changes in concentration of TNF vary by host time-of-day and dose?

**Fig. 3:**
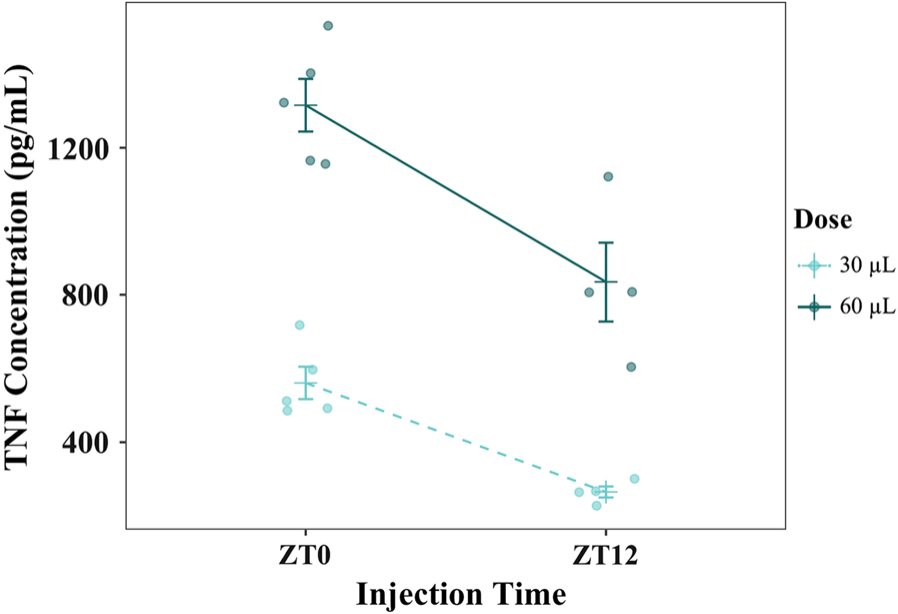
TNF concentrations (pg/ML) alongside mean ± SE at 2 HPI, after treatment at either ZT0 (lights-on) or ZT12 (lights-off) for two doses of TNF (30 μg/kg or 60 μg/kg). Points represent raw data

Dose (P < 0.0001) and host time-of-day (P < 0.001) contributed substantially to TNF concentration 2 HPI (Fig. 3) but there was no interaction between them (P = 0.192). The concentration of TNF in mice that were injected at ZT12 (i.e. lights-off, entering the active phase) was 388.73 pg/mL (± 112.88) lower at 2 HPI than in those mice injected at ZT0 (i.e. lights-on, entering the resting phase) (Fig. 3).

#### Is gametocyte vulnerability to TNF dependent on host time-of-day?

**Fig. 4:**
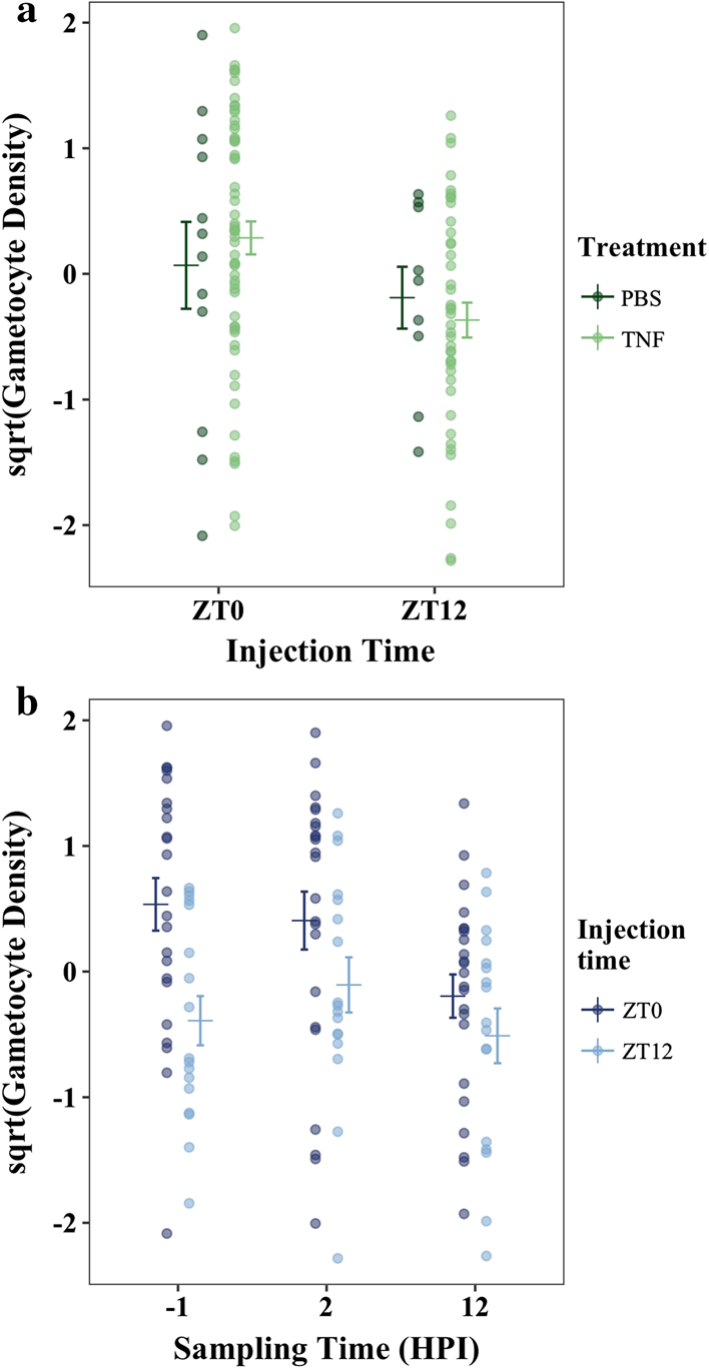
**a** Cumulative gametocyte density (gametocytes/mL blood) for all sampling timepoints, alongside mean ± SE (calculated post-transformation), in mice injected with either TNF or carrier at either ZT0 or ZT12. **b** Gametocyte density (gametocytes/mL blood), alongside mean ± SE, for combined treatment groups (TNF and control) in mice injected at either ZT0 or ZT12 for each sampling time (− 1 (baseline), 2-, and 12 HPI). In both plots, points represent raw data, square-root-transformed to approximate normality, and scaled to have a mean of 0 and a standard deviation of 1

No evidence was found to support the interaction between host time-of-day (i.e. injection time) and TNF on gametocyte density (Fig. 4a; Table 2). Indeed, the most parsimonious model included only host time-of-day (injection time ZT0 or ZT12) and sampling time (2- or 12-HPI) as main effects (ΔAICc = 0; Table 2; Fig. 4b). The model incorporating only sampling time was within 2 ΔAICc (ΔAICc = 1.588; Table 2) and is therefore competitive with the most parsimonious model. Furthermore, the most parsimonious model returned only a ~ 48% chance of being the best approximating model in the given model set (AICc weight = 0.482, Table 2), indicating high model selection uncertainty. Notably, including treatment (TNF or PBS) reduced model fits (Table 2). Overall, this analysis finds no evidence to suggest that TNF or injection time (i.e. host time-of-day) effect gametocyte density.

**Table 2:** Degrees of freedom (df), log-Likelihood (log(*L*)), AICc, ΔAICc (AICc_*i*_ − AICc_*min*_), and AIC *w* (AICc weight) for each linear model in the TNF ii analysis ordered in descending fit

Model description: sqrt(gametocyte density) ~ + (1|mouse)	df	log(*L*)	AICc	ΔAICc	AICc *w*
Inj.time + samp.time	6	− 137.7	288.1	0.000	0.482
Samp.time	5	− 139.6	289.7	1.588	0.218
Inj.time + samp.time + treatment	7	− 137.8	290.6	2.497	0.138
Inj.time + samp.time + treatment + inj.time*treatment	8	− 137.1	291.4	3.318	0.092
Samp.time + treatment	6	− 139.7	292.1	3.953	0.067
Inj.time	4	− 145.3	299.0	10.872	0.002
Null	3	− 147.2	300.6	12.536	0.001
Inj.time + treatment	5	− 145.4	301.4	13.291	0.001
Treatment	4	− 147.3	302.9	14.825	0.000

The response variable for each model is the square root-transformed gametocyte density and the random effect is “mouse”. “Samp.time” refers to sampling time (2- or 12-HPI), “inj.time” refers to injection time (ZT0 or ZT12 respectively), and treatment is either TNF-α or control. The null model includes only “mouse” as a random effect

#### Is gametocyte vulnerability to TNF dependent on gametocyte age?

**Fig. 5:**
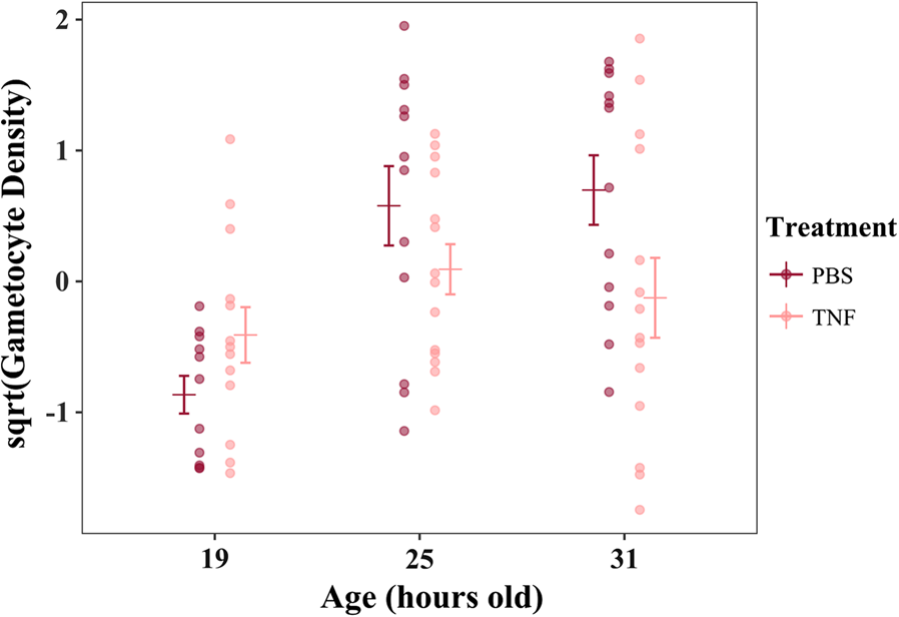
Gametocyte density (gametocytes/mL blood), alongside mean ± SE (calculated post-transformation), averaged across all sampling timepoints (4-, 8-, and 12-HPI) for gametocytes treated at ages 19-, 25-, and 31-h old and according to whether they were treated with TNF or PBS carrier only. Points represent raw data, square root-transformed to approximate normality, and scaled to have a mean of 0 and a standard deviation of 1

The most parsimonious model included only gametocyte age (ΔAICc = 0; Table 3), but evidence for this being the best fitting model is weak (AIC *w *= 0.409; Table 3). The two next most competitive models included age and treatment (ΔAICc = 0.850; Table 3) and age, treatment, and the treatment by age interaction (ΔAICc = 1.785; Table 3). Because the treatment by age interaction is only present in one of the competing models, and the AICc weight for this model is very low (AICc *w *= 0.267; Table 3), it is unlikely that this parameter is important in explaining gametocyte density. Thus, in keeping with the results of experiment ii, there is no clear evidence to support a role for TNF in differentially affecting gametocytes of varying ages (Fig. 5; Table 3).

**Table 3:** Degrees of freedom (df), log-Likelihood (log(*L*)), AICc, ΔAICc (AICc_*model*_ − AICc_*min model*_), and AICc *w* for each linear model in the analysis of experiment iii ordered in descending fit

Model description: sqrt(Gametocyte Density) ~ + (1|mouse)	df	log(*L*)	AICc	ΔAICc	AICc *w*
Age	5	− 99.37	209.6	0.000	0.409
Age + treatment + age * treatment	8	− 96.15	210.4	0.850	0.267
Age + treatment	6	− 99.08	211.4	1.785	0.168
Null	3	− 103.5	213.3	3.759	0.062
Age + samp.time	7	− 99.52	214.7	5.088	0.032
Treatment	4	− 103.3	215.1	5.469	0.027
Age + samp.time + treatment + age*treatment	10	− 96.38	216.1	6.556	0.015
Age + samp.time + treatment	8	− 99.22	216.6	7.000	0.012
Samp.time	5	− 103.7	218.3	8.743	0.005
Samp.time + treatment	6	− 103.5	220.2	10.57	0.002

The response variable for each model is the square root-transformed gametocyte density and the random effect is “mouse”. “Samp.time” refers to sampling time (4-, 8- or 12-HPI), “age” refers to gametocyte age (19-, 25-, or 31-h old) and treatment is either TNF-α or control. The null model includes only “mouse” as a random effect

## Discussion

The experiments presented here suggest that neither the conversion nor the immune killing hypotheses can explain the reduction in gametocytes when parasites are out-of-synch with host circadian rhythms. First, parasites were predicted to reduce conversion in out-of-synch infections to reduce the impact of being out-of-synch on asexual densities. However, average conversion rates in the dataset that met strict model selection criteria (Petra PLoS Path) and also the full dataset of all infections (Additional file 1) did not vary significantly between in- and out-of-synch infections (Table 1, Fig. 2, Additional file 1). Second, TNF (for both doses) introduced at the start of the resting phase (i.e. lights-on) was cleared at a slower rate compared to the start of the active phase (i.e. lights-off, experiment (i). This time-of-day effect is likely explained by temporal variation in host metabolic rates: heightened metabolism during the active phase should clear incoming TNF more readily than the during the rest phase. Despite evidence for host circadian rhythms influencing TNF levels, which is consistent with other studies [21, 29, 30], two experiments (ii) and (iii); Figs. 4, 5; Tables 2, 3) suggest that exposure to more TNF, or for longer, have no significant impact on the densities of gametocytes, even when exposed at different ages.

The conversion results suggest that either parasites do not adjust their conversion rate when out-of-synch with host rhythms, or that the methods utilised here were unable detect change. Conversion is a key determinant of transmission potential (thus, fitness) and a phenotypically plastic trait. Conversion is reduced by *P. chabaudi* in response to a loss of “state” (i.e. reduction in density or replication) [10] so parasites would not need to detect that they are out-of-synch but simply respond to the impact of being out-of-synch upon state. It is possible that the modest drop in asexual density in the first couple of days post-infection [6] is not sufficiently stressful to elicit reproductive restraint [10]—particularly because out-of-synch parasites still experience high replication rates despite being at a lower initial density. In addition, the data and approach used to test the conversion hypothesis were likely conservative. For example, there may be a minimal level of change in the conversion rate is required for the approach of Greischar et al. [22] to reliably return different conversion estimates, and this level may not have been met. When out-of-synch with host rhythms, parasites begin the process of rescheduling to regain coordination between the IDC and host rhythms [31]. Whether rescheduling affects the gametocyte developmental schedule is unknown, but variation in how gametocytes accumulate over time could compromise the reliability of conversion estimates. It is, therefore, possible that when out-of-synch with host rhythms, parasites do reduce conversion accordingly but more severe perturbations are required to detect this. For example, future work might compare conversion of in- and out-of-synch parasites when also exposed to in-host competition or resource limitation.

That TNF rhythms and the age of gametocytes had no significant effect on gametocyte density was unexpected. Schizogony (the production of a new cohort of asexuals and gametocytes) causes an elevation of TNF that may be capable of sterilizing gametocytes [4]. The concentration of TNF used in experiment ii is greater than schizogony-induced TNF levels, and should therefore represent a meaningful change to the within-host environment. However, time-of-day-dependent clearance of TNF may have resulted in confounding host and parasite time-of-day in experiment ii. Experiment iii corrected for this by directly testing gametocyte age without host time-of-day as a confounding factor (via the use of circadian-knockout mice), although it was impossible to eliminate TNF that was produced as a result of schizogony. It remains uncertain how schizogony-induced TNF might have affected the focal gametocyte cohorts, but it may have acted either directly by attacking gametocytes, or indirectly by activating related immune cells or factors involved in clearing TNF. Further, gametocyte numbers are generally low, rendering them extremely difficult to detect, particularly where minor perturbations to density may have been confounded by other within-host rhythms. Why blocking the TNF receptor results in an increase in gametocyte density [19], yet introducing TNF does not appear to decrease gametocyte density, remains mysterious. One possibility is that downstream immune cells activated by TNF are responsible for reducing gametocyte density, and in the absence of TNF these cells remain inactive. In this scenario, introducing more TNF (as done here) may have little to no additional impact at all on gametocyte density if the threshold for activation of these immune cells is low.

To address the potentially confounding effects of schizogony-induced TNF, future work could aim to block TNF (following [19]) in circadian-knockout mice at the onset of schizogony. The effect of rhythmic TNF on gametocyte survivability (replicated by artificial injection) could then be better understood without the presence of potentially opposing rhythms. Additionally, other rhythmic host resources required for gametocyte development such as LysoPC (host-derived lipid) [32] may be limited in out-of-synch infections. Repeating a similar experiment to experiment iii but using resources essential to gametocyte development could elucidate whether the gametocyte density decrease in out-of-synch infections can be attributed to host offensive rhythms, or whether gametocyte development and survival is passively modulated through host circadian processes (e.g. the availability of nutrients). Further, out-of-synch gametocytes may alter their developmental rate to reschedule to match host circadian rhythms, accounting for some of the observed reduction in gametocyte density. Simultaneously considering the multitude of factors contributing to gametocyte reduction could provide an explanation for the substantial decrease in gametocytes in out-of-synch infections.

## Conclusion

Understanding consequences of being out-of-synch with host circadian rhythms is important for unravelling the evolutionary drivers of rhythmic development in malaria parasites. Knowledge of the benefits, or costs, to parasites of being in-synch with host rhythms will allow them to be harnessed for the development of novel anti-malarial treatments; for example, if gametocytes show time-of-day-specific vulnerabilities, drugs could take advantage of rhythmic weaknesses. Further, understanding how malaria parasites respond to temporal variation in the within-host environment is increasingly important as some mosquito populations are reported to have shifted the timing of blood-foraging rhythms in response to the widespread use of long-lasting insecticide-treated bed nets [33, 34]. Since gametocytes are necessary for transmission, understanding what causes their reduction in out-of-sync infections could have implications for shifts in vector rhythms. If out-of-synch parasites are less fit, changes in vector behaviour could be beneficial in minimizing malaria burden. However, without fully understanding the evolutionary drivers behind parasite rhythms it is difficult to predict how, or if, parasites might evolve to cope with these changes.

## Supplementary information

**Additional file 1.** Additional table and figure.

## Data Availability

The datasets used and/or analysed during the current study are available from the corresponding author on reasonable request.

## Abbreviations

IDC: intraerythrocytic developmental cycle
TNF: tumour necrosis factor
PCNA1: proliferating‐cell nuclear antigen 1
WT: wild-type
RBC: red blood cell
PABA: para-aminobenzoic acid
PHZ: phenylhydrazine
PI: post infection
PBS: phosphate-buffered saline
ZT: zeitgeber time
GMT: greenwich Mean Time
IP: intraperitoneal
ELISA: quantitative enzyme-linked immunosorbent assay
HR: hour
AICc: akaike information criterion-corrected
SE: standard error
