# Supplementary material for: Testing possible causes of gametocyte reduction in temporally out-of-synch malaria infections
Source: Malar J. 2020 Jan 14;19:17. doi: 10.1186/s12936-020-3107-1 (PMC6958767; doi:10.1186/s12936-020-3107-1)
Supplement: Supplementary file 1 — Additional file 1. Additional table and figure. [file 12936_2020_3107_MOESM1_ESM.docx]

**Additional File 1**

| Model description:  Log10(Conversion) ~ | df | log(*L*) | AICc | ΔAICc | AICc *w* |
| --- | --- | --- | --- | --- | --- |
| donor | 3 | -13.80 | 34.80 | 0.000 | 0.648 |
| donor + schedule | 4 | -13.77 | 37.64 | 2.842 | 0.156 |
| null | 2 | -16.74 | 38.05 | 3.252 | 0.127 |
| schedule | 3 | -16.72 | 40.63 | 5.831 | 0.035 |
| donor + schedule + donor*scheule | 5 | -13.71 | 40.75 | 5.950 | 0.033 |

Additional File 1 Table. Degrees of freedom (df), log-Likelihood (log(*L*)), AICc, ΔAICc (AICc*_i_* – AICc*_min_*), and AICc *w* (AICc weight) for each linear model in the conversion analysis using the full dataset ordered in descending fit (best-fitting model at the top). The response variable for each model is the log_10_-transformed conversion rate. “Schedule” refers to parasites either in-synch or out-of-synch with the host, and “donor” corresponds to parasites taken from donor mice kept in either the standard or reversed light : dark schedule.


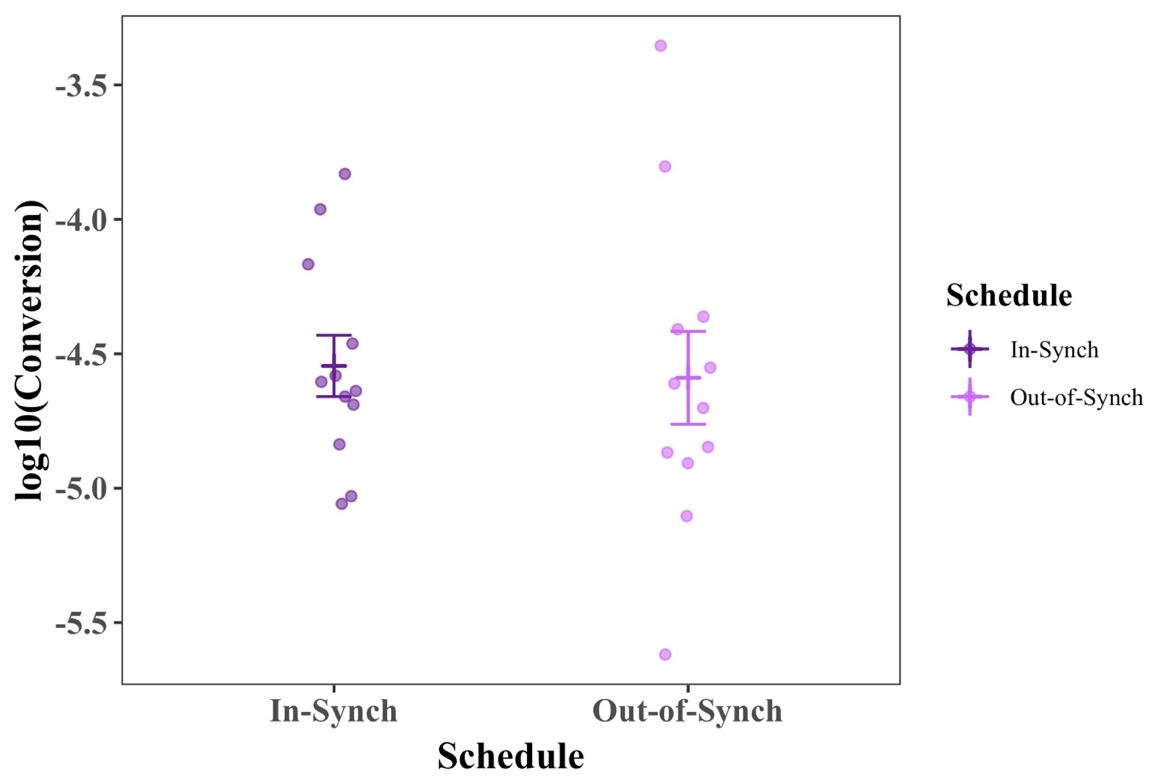


Additional File 1 Figure. Conversion estimates, alongside mean ± S.E. (calculated post-transformation), for parasites in- and out-of-synch with host circadian rhythms using the full dataset. Points represent raw data, log_10_-transformed to approximate normality. Data from experiment in O’Donnell et al. 2011.
